# Dosimetric Comparison of Intensity-Modulated Radiation Therapy (IMRT) and Intensity-Modulated Proton Therapy (IMPT) for a Novel Oral Tongue Avoidance Concept in Low-Risk Squamous Cell Carcinoma of the Oral Tongue

**DOI:** 10.14338/IJPT-22-00032

**Published:** 2023-02-16

**Authors:** Robert H Press, Lei Hu, Sheng Huang, Shaakir Hasan, J. Isabelle Choi, Charles B. Simone, Arpit M. Chhabra, Daphna Y. Gelblum, Rafi Kabarriti, Richard L. Bakst, Jen R. Cracchiolo, Sean M. McBride, Nancy Y. Lee

**Affiliations:** 1 New York Proton Center, New York, NY, USA; 2 Department of Radiation Oncology, Memorial Sloan Kettering Cancer Center, New York, NY, USA; 3 Department of Radiation Oncology, Albert Einstein College of Medicine and Montefiore Medical Center, Bronx, NY, USA; 4 Department of Radiation Oncology, Icahn School of Medicine at Mount Sinai, New York, NY, USA; 5 Department of Surgery, Memorial Sloan Kettering Cancer Center, New York, NY, USA

**Keywords:** oral tongue, oral cavity, dosimetric analysis, proton therapy

## Abstract

**Purpose:**

After adequate surgical resection, early-stage oral tongue cancer patients can harbor a low risk of local recurrence but remain at risk of regional recurrence. Oral tongue avoidance during adjuvant radiation therapy is an attractive potential treatment strategy to mitigate treatment toxicity. We sought to quantify the dosimetric advantages of this approach and hypothesized that intensity-modulated proton therapy (IMPT) may further reduce organs at risk doses compared with intensity-modulated radiation therapy (IMRT).

**Materials and Methods:**

Five patients with oral tongue cancer treated with postoperative radiation therapy from August 2020 to September 2021 were retrospectively reviewed. Novel clinical target volume contours, excluding the oral tongue, were generated while maintaining coverage of bilateral at-risk lymph nodes. Comparison IMRT (X) and IMPT (PBT) plans were generated using standard treatment volumes (control) and avoidance volumes (study) (n = 4 plans/patient). Dosimetric variables for organs at risk were compared using the paired *t* test.

**Results:**

The prescribed dose was 60 Gy in 30 fractions. D95% clinical target volume coverage was similar between X and PBT plans for both control and study clinical target volumes. Comparing control with study plans, both X (58.9 Gy vs 38.3 Gy, *P* = .007) and PBT (60.2 Gy vs 26.1 Gy, *P* < .001) decreased the oral cavity dose_mean_. The pharyngeal constrictor dose_mean_ was also reduced (*P* < .003). There was no difference between control and study plans for larynx (*P* = .19), parotid (*P* = .11), or mandible dose (*P* = .59). For study plans, PBT significantly reduced oral cavity dose_mean_ (38.3 Gy vs 26.1 Gy, *P* = .007) and parotid dose_mean_ (23.3 Gy vs 19.3 Gy, *P* = .03) compared with X. For control plans, there was no difference in oral cavity dose_mean_ using PBT compared with X, but PBT did improve the parotid dose_mean_ (26.6 Gy vs 19.7 Gy, *P* = .02).

**Conclusion:**

This study quantifies the feasibility and dosimetric advantages of oral tongue avoidance while still treating the at-risk lymph nodes for oral tongue cancer. The dosimetric difference between PBT and X was most prominent with an oral tongue–avoidance strategy.

## Introduction

Oral cavity cancer is a challenging disease entity that often necessitates highly morbid multimodality treatment to achieve a cure. Incidence has increased over the last 20 years, with an approximate rate of 3.6 per 100 000, resulting in nearly 18 000 cases diagnosed and nearly 3000 deaths annually [1]. The standard early-stage node-negative oral tongue cancer treatment includes partial glossectomy with an ipsilateral elective neck dissection [2]. Utilization of elective neck dissection is often driven by the depth of invasion (DOI) because DOI greater than 4 mm is associated with a greater than 20% risk of regional failure [3].

In cases of increased DOI and/or other pathologic risk factors, such as perineural invasion, lymphovascular invasion, or close or positive margins, postoperative radiation therapy is used to reduce the risk of locoregional recurrence. Adjuvant radiation therapy typically targets the entire oral tongue, bilateral elective neck levels, and in-transit lymphatics due to the risk of skip metastases. For tumors with significant DOI or clinically positive ipsilateral neck disease, the contralateral neck can remain at risk of nodal failure even in lateralized tumors [4,5]. While considered the standard of care, intentional irradiation of the oral tongue can be particularly toxic and result in acute and late toxicities, including confluent mucositis, persistent dysgeusia, swallowing dysfunction, speech alteration, osteoradionecrosis, and dental caries [6].

The pattern of failure for patients with low-risk node-negative oral tongue cancer can vary depending on certain risk factors. Close and/or involved surgical margins are associated with in-tongue recurrences [7], whereas perineural invasion, often thought of as a surrogate for close margins [8], has also been associated with regional and distant failure [9]. A DOI between 5 and 10 mm, lymphovascular invasion, and node positivity are also associated with locoregional risk as the primary pattern of failure [10–12].

In select patients with a low risk of in-tongue local failure but indications for adjuvant radiation therapy due to the risk of regional failure (eg, DOI > 5 mm and/or node-positive disease), the contribution of primary site irradiation to disease control is unclear. Avoiding irradiation of the oral tongue in this select group is expected to greatly mitigate treatment morbidity. Prior studies avoiding irradiation to the primary site after adequate surgical resection for other head and neck cancer subsites, such as oropharyngeal cancer, have been successfully reported [13,14]. Therefore, an investigator-initiated trial to test the safety and efficacy of oral-tongue avoidance in low-risk oral tongue cancer patients is currently being developed. Before the initiation of this trial, we sought to assess the feasibility and quantify the dosimetric advantage of this strategy, hypothesizing that significant improvements in doses to organs at risk (OARs) would be achieved when avoiding the oral tongue. In addition, the use of intensity-modulated proton therapy (IMPT) for this strategy may accentuate sparing OARs compared with intensity-modulated radiation therapy (IMRT).

## Materials and Methods

This study was approved by the New York Proton Center institutional review board, and a waiver of informed consent was granted because of the retrospective nature of the study. Five oral tongue cancer patients who received adjuvant proton beam therapy (PBT) at the New York Proton Center from August 2020 to September 2021 were identified. Patients were treated using pencil-beam scanning IMPT targeting a standard clinical target volume (CTV), including the postoperative bed and oral tongue remnant, at-risk bilateral neck levels I–IV, and in-transit lymphatics. Primary site volumes were defined per our institutional standard incorporating preoperative imaging and exam, operative findings, and pathologic findings. Nodal levels IA, IB, II, III, and IV and OARs were contoured per consensus guidelines [15,16]. The oral tongue and dissected neck were prescribed to 60 Gy in 30 fractions, while the undissected contralateral neck received 54 Gy in 30 fractions using a simultaneous integrated boost. Comparison IMRT plans were generated for each patient and optimized to the identical CTV plus a 3-mm planning target volume margin with similar goal parameters for target coverage and OAR sparing. For this study, plans using standard target volumes were considered control plans.

**Figure 1.**
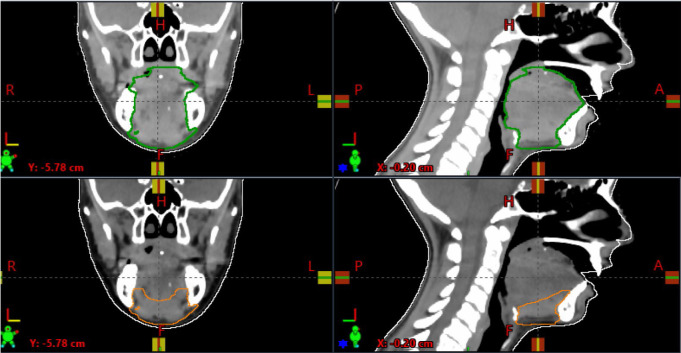
Representative target contours for control (green, top row) and study (orange, bottom row) plans.

Next, experimental CTV contours were created that excluded the oral tongue while maintaining coverage of at-risk lymph nodes, intra-transit lymphatics, and the floor of the mouth to the superior border of the hyoid bone (**Figure 1**). Plans using the experimental target volumes were considered study plans. Target volumes were independently verified for each patient by 2 radiation oncologists (RHP, NYL) before study plan optimization. Next, IMRT and IMPT plans were generated using the new study volumes.

All IMPT planning used a 5-beam multifield optimization technique with 2 posterior oblique fields, 2 anterior oblique fields, and a posterior-anterior field. Field-specific targets were generated for each beam, and an individual field simultaneous optimization technique was applied. Plans were developed using robust optimization of the target CTV with 3 mm setup uncertainties in the x, y, and z directions and ± 3.5% range uncertainty. An additional 3-mm field of uncertainty in the superior-inferior direction was included to ensure a smooth dose transition between fields covering the superior and inferior regions of the target. Both spot spacing and layer spacing were 5 mm. Intended CTV coverage was D95% ≥ 100%. A density to water equivalence was performed for the dental artifact. The plans were robustly optimized using the Eclipse treatment planning system (Varian Medical Systems, Palo Alto, CA). A dose in Gy was prescribed using a relative biological effectiveness of 1.1.

All IMRT planning was also performed in the Eclipse treatment planning system, using 6-MV photon beams and 5-mm width multileaf collimators (Millennium 120 leaf). A volumetric modulated arc therapy technique was applied, with 2 full arcs of 90° collimator rotation in relation to each other. Plans were targeted to a planning target volume, created with a 3-mm expansion on the CTV, with equivalent intended CTV coverage of D95% ≥ 100% comparable to the IMPT planning.

In total, each patient had 4 plans for comparison (PBT Control, IMRT Control, PBT Study, and IMRT Study). Dosimetric variables for critical OARs, including the oral cavity, parotid glands (ipsilateral and contralateral), pharyngeal constrictor muscles, larynx, and mandible, were compared using the paired *t* test. Statistical analysis was performed using SPSS version 20 (IBM, Armonk, NY) and Excel version 2010 (Microsoft, Redmond, WA). A *P* value < .05 was considered statistically significant.

## Results

The median age of the patient cohort was 70 years (range 27-80). All patients had pT1-2 and pN0-N2b disease with complete margin-negative partial or hemiglossectomy and ipsilateral neck dissections. Overall, plans were normalized such that D95% CTV coverage was similar between IMRT and PBT plans for both control and study target volumes (all *P* ≥ .98). Full dose comparisons for control and study plans are listed in the **Table**.

**Table.** Dose comparison between photon therapy and proton beam therapy (PBT).

Patient	Therapy	CTV_6000 D95% (%)	Oral cavity, mean (cGy)	Pharyngeal constrictor, mean (cGy)	Larynx, mean (cGy)	Parotid ipsilateral, mean (cGy)	Parotid contralatera, mean (cGy)	Mandible, dmax (cGy)
Control plan								
1	Photon	99.6	6128	5445	5605	1795	2345	6531
	PBT	99.0	6174	4924	4477	1568	2400	6523
2	Photon	99.8	6159	5200	4576	4387	2266	6406
	PBT	99.4	6167	4889	3097	3155	787	6517
3	Photon	96.7	5813	3318	3673	3072	1972	6616
	PBT	99.5	6223	4453	5292	2442	1508	6307
4	Photon	100.5	5531	4964	4834	2359	2062	6233
	PBT	100.4	5728	4761	4240	1425	1543	6493
5	Photon	100.7	5816	4944	4550	1666	2631	6543
	PBT	99.0	5824	3456	3978	1270	2385	6375
Photon mean		99.5	5889	4774	4648	2656	2255	6466
PBT mean		99.5	6023	4497	4217	1972	1725	6443
*t* test, *P*		.67	.16	.54	.47	**.019**	.12	.83
Study plan								
1	Photon	100.0	4993	5269	5460	1814	2367	6507
	PBT	99.1	3028	4284	4536	1554	2475	6521
2	Photon	98.0	4560	4970	4645	3962	1914	6475
	PBT	99.6	3556	4023	3005	3115	591	6718
3	Photon	100.8	4018	2939	3210	2622	1967	6447
	PBT	98.1	2770	3100	3815	2471	1391	6165
4	Photon	100.5	2063	4541	4883	1627	1529	6247
	PBT	100.4	1564	3643	4196	1159	1138	6531
5	Photon	100.6	3517	4567	4289	1642	2635	6534
	PBT	99.0	2114	2378	3999	1354	2476	6429
Photon mean		100.0	3830	4457	4497	2333	2082	6442
PBT mean		99.2	2606	3486	3910	1931	1614	6473
*t* test, *P*		.98	**.007**	.059	.19	**.03**	.12	.79

**Abbreviation:** CTV, clinical target volume.

Note: Bolded values are statistically significant.

Comparing control with study plans, both IMRT (58.9 Gy vs. 38.3 Gy, *P* = .007) and PBT (60.2 Gy vs 26.1 Gy, *P* < .001) study plans demonstrated significant reductions in the oral cavity mean dose. Similar results were demonstrated for pharyngeal constrictor mean dose for both IMRT (47.7 Gy vs 44.6 Gy, *P* = .003) and PBT (45.0 Gy vs 34.9 Gy, *P* = .001) study plans. There was no difference between the control and study plans for the larynx (*P* > .19) or parotid (*P* > .11) mean dose or the mandible max dose (*P* > .59).

For Control plans, there was no difference in dose to the oral cavity, pharyngeal constrictors, larynx, or mandible between PBT and IMRT plans. PBT did, however, result in an improvement in mean ipsilateral parotid dose (26.6 Gy vs 19.7 Gy, *P* = .02, whereas the contralateral parotid mean dose was not significantly different (20.8 Gy vs 16.1 Gy, *P* = .12).

**Figure 2.**
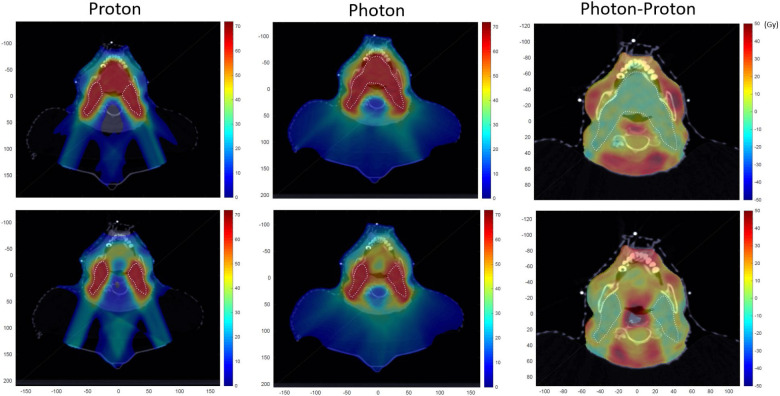
Representative axial dose distribution of proton and photon plan for the control and study groups. The dose difference (photon–proton) are also presented in the last column.

Comparing PBT and IMRT study plans, PBT resulted in further reduction in the oral cavity mean dose (38.3 Gy vs 26.1 Gy, *P* = .007) and reduction in the ipsilateral parotid mean dose (23.3 Gy vs 19.3 Gy, *P* = .03) compared with IMRT. The pharyngeal constrictor mean dose trended toward improvement (44.6 Gy vs 34.9 Gy, *P* = .059), while the larynx and contralateral parotid mean dose and mandible max dose were not significantly different. The dose differences between control and study plans are demonstrated in a representative dose wash (**Figure 2**).

## Discussion

Adjuvant radiation therapy for oral tongue cancer can be particularly morbid. This is primarily driven by irradiation of the mobile oral tongue, which can result in florid mucositis, dysgeusia, and dysphagia. Conventional radiation therapy target volumes for early-stage oral tongue cancer are controversial [17], and there is interest in an oral tongue avoidance strategy for select low-risk patients. The current study sought to quantify the dosimetric advantage of this strategy.

As expected, the omission of the mobile oral tongue from radiation therapy target volumes significantly minimizes oral cavity dose, resulting in a roughly 50% relative reduction in the mean dose. This is apparent for both IMRT and PBT treatment plans. There was also a modest but significant reduction in the pharyngeal constrictor mean dose. While the oral cavity dose for standard target volumes is equivalent between PBT and IMRT, the current study demonstrates the use of PBT for oral cavity avoidance enables an additional 30% relative reduction in oral cavity mean dose compared with IMRT, as well as modest improvements in ipsilateral parotid gland mean dose. PBT, therefore, appears well suited for this strategy by exploiting the Bragg Peak phenomenon to stop dose contribution from posterior beams required for coverage of high-level II and retrostyloid nodal regions into the oral cavity.

The clinical significance of this dose reduction still requires further investigation. Studies of long-term toxicity after definitive treatment for oral tongue cancers report that up to 40% of patients developed late toxicities, and up to 50% and 33% of patients were opioid dependent 3 and 6 months after completion of therapy, respectively [6]. Meanwhile, validated normal tissue complication probability models suggest that reducing the mean oral cavity dose from 38 to 26 Gy would reduce the risk of grade 3+ mucositis from 42% to 34% [18]. In addition, comparative analyses of PBT and IMRT for oropharyngeal cancers, a similar treatment scenario intended to avoid dose to the oral cavity, corroborates these models, reporting PBT resulted in lower percutaneous endoscopic gastrostomy placement rates (odds ratio 0.27), less acute hospitalizations, and improved patient-reported outcomes including narcotic use and dysgeusia compared with modern photon radiation therapy techniques [19,20].

As surgical techniques improve with advances, such as free tissue reconstruction and improved margin assessment, the risk of local recurrence at the primary site may be decreasing, and attempts to avoid intentional irradiation of the primary site is appealing to widen the therapeutic ratio by deintensifying treatment paradigms. This strategy is already being implemented in early-stage oropharyngeal cancer treated with upfront transoral robotic surgery resection. A recent single-arm phase II trial from the University of Pennsylvania reported that the omission of resected primary tumor bed irradiation resulted in a 2-year local control of 98.3%, only a 3.3% rate of late soft tissue necrosis, and no patients with long-term dependence on feeding tubes [13]. Of note, in this study, the mean radiation dose to the primary site was 40 Gy, similar to the achieved mean dose to the entire oral cavity from IMRT in our study. Another recent prospective study from the Mayo Clinic using primarily PBT to exclude the primary mucosal site treated 64 patients and reported a 2-year local control of 98.4% and significant improvements in patient-reported toxicity domains, including pain, swallowing, senses, speech, contact, and mouth opening. Only 2 patients on trial required percutaneous endoscopic gastrostomy tube placement due to poor oral intake and dysgeusia, both of whom were treated with IMRT due to PBT insurance denial. The primary mucosal site mean dose in this study was 9.6 Gy, drastically reduced compared with the University of Pennsylvania study that used IMRT [14]. These results further corroborate our dosimetric analysis that PBT can further reduce oral mucosal dose compared with IMRT and suggest possible gains in clinical toxicity outcomes.

While intriguing, an oral tongue avoidance strategy needs to be implemented cautiously and in a prospective clinical trial with standardization and quality assurance of experimental target volumes. Salvage surgery for recurrent oral cavity cancer is often inadequate and associated with significant morbidity and mortality [21]. Other risk factors should be considered to optimize patient selection for those who are at low risk of local recurrence, such as the patient's age, possible extent of the tumor into the floor of the mouth or other oral cavity subsite, adequacy of the surgical resection or nodal dissection, and the worst pattern of invasion on pathology.

This study has specific limitations. Target volume delineation in oral cavity cancer is controversial. For example, primary tumor size and/or oral cavity location could greatly influence target volumes (eg, ipsilateral nodal irradiation, avoidance of level IA) and subsequently alter dosimetric outcomes. The current study only evaluated patients with oral tongue primary cancers treated with bilateral nodal irradiation. A single institution also performed IMRT and IMPT planning methods, and different optimization and delivery methods may further affect these results.

In conclusion, omission of intentional irradiation to the mobile oral tongue for low-risk oral tongue cancer treated with adequate surgical resection can still allow for full coverage to the remainder of the target volume and results in clear dosimetric improvements in OAR dose, particularly for the oral cavity and pharyngeal constrictor muscles. PBT for this strategy enhances these improvements, and normal tissue complication probability models indicate a clinically meaningful reduction in toxicity. These results serve as the basis for an investigator-initiated trial evaluating the clinical efficacy of oral tongue avoidance for favorable oral tongue cancer patients with indications for elective nodal irradiation that is under development, and the inclusion of PBT as part of this trial paradigm is warranted based on the demonstrated dosimetric advantage.
